# Editorial: Memory Systems of the Addicted Brain: The Underestimated Role of Cognitive Biases in Addiction and Its Treatment

**DOI:** 10.3389/fpsyt.2018.00030

**Published:** 2018-02-19

**Authors:** Vincent David, Daniel Béracochéa, Mark E. Walton

**Affiliations:** ^1^Institut de Neurosciences Cognitives et Intégratives d’Aquitaine, CNRS UMR 5287, Pessac, France; ^2^Université de Bordeaux, Pessac, France; ^3^Department of Experimental Psychology, University of Oxford, Oxford, United Kingdom

**Keywords:** addiction, memory systems, decision-making, habit learning, alcohol

**Editorial on the Research Topic**

**Memory Systems of the Addicted Brain: The Underestimated Role of Cognitive Biases in Addiction and Its Treatment**

Drug addiction has often been viewed as an aberrant form of learning during which strong associations linking actions to drug seeking are expressed as persistent stimulus–response habits, thereby maintaining a vulnerability to relapse. However, an increasing body of data suggests a more complex picture, revealing that different cognitive processes are altered by drug use or abuse. These alterations clearly need to be taken into account to better understand addictive behaviors, as they are likely to contribute to their persistence and their response to pharmacological and non-pharmacological treatments. Therefore, the aim of this research topic is to provide an overview of the current work investigating the long-term impact of drug use on learning, memory, and decision-making processes, how multiple memory systems modulate drug-seeking behavior, as well as how drug-induced cognitive biases could contribute to the persistence of addictive behaviors. Another interesting feature of this research topic is that new animal models of cognitive processes pertaining to addictions are presented, providing strong support to the translational interest of these tasks.

The research topic begins with a commentary repositioning the initiative on precision medicine launched by the National Institutes of Health in the context of addictions (Ghitza), and a comprehensive presentation of neuropsychological consequences of chronic drug use across a wide range of different substances (Cadet and Bisagno). The emerging picture is that drugs of abuse have effects on cognitive processes which go far beyond their well-known habit forming action. In fact, under certain circumstances, evidence now exists that repeated cocaine exposure appears to *promote* more complex goal-directed behaviors (Halbout et al.).

Chronic drug use increasingly appears to have also long-lasting effects on interactions between memory systems, which are a normal aspect of learning. Both human and rodent studies support the view that the hippocampus and the dorsal striatum can interact in either a cooperative or a competitive manner during learning, with the prefrontal cortex being involved in the selection of an appropriate learning strategy. Building on original studies of Norman M. White 20 years ago, a comprehensive review describes how chronic consumption of drugs of abuse impacts normal interactions between these memory systems (Goodman and Packard). Within this theoretical framework, an experimental report further shows that opiate self-administration eventually leads to a functional imbalance characterized by an exclusive use of striatum-dependent learning strategies, at the expense of hippocampal-dependent processes, in rodents performing navigational tasks (Baudonnat et al.). One structure that may be critical for acting as a switch between memory systems, the ventral tegmental area, is the focus of an in-depth review looking closely into its afferent circuits and their specific implication into drug-related behaviors (Oliva and Wanat).

In the recent version of the Diagnostic and Statistical Manual of Psychiatric Disorders (DSM V), alcohol and drug addiction have been combined under the new classification of substance use disorders. Common behavioral symptoms with diagnostic value now include already existing criteria such as loss of control, negative affect upon withdrawal, vulnerability to relapse and lately, craving defined as an urgent desire to use the target substance. In the present issue, several reviews and experimental studies present compelling evidence that alcohol abuse lead to long-lasting changes in learning processes, which may contribute to persistent alcoholism (Corbit and Janak; Staples and Mandyam). Nicely complementing the description of these behavioral changes, other authors have reviewed extensively what is currently known about the role of epigenetic marks (histone deacetylation) in the glucocorticoid-dependent dysregulation of the hypothalamic–pituitary–adrenal axis activity (Mons and Beracochea).

A loss of cognitive flexibility may also be observed through assessment of decision-making processes, an essential component of our daily life. They may be uncovered by imposing rule changes on the subject, such as requiring an attentional shift between different perceptual features of a complex stimulus, as in the attentional set shifting task which was recently adapted to rodents (Besson and Forget). In this issue, Granon and colleagues (Pittaras et al.) provide evidence to implicate β2 nicotinic receptors in the excitation/inhibition balance in the prefrontal cortex using β2^−/−^ mice, which exhibit inappropriate decision-making and a blunted sensitivity to punishment when outcome uncertainty is high. These reports are especially interesting in that they also provide new means to evaluate carefully decision-making in rodents.

The importance of a better understanding, at both the experimental and theoretical levels, of decision-making processes for the purpose of addiction treatments is highlighted by the study of Regier and Redish on contingency management. The authors challenge the view that the success of this approach relies solely on alternative reinforcement. Instead, they provide evidence that access to deliberative decision-making processes, and bypass of automatic action-selection systems, may be the key to the therapeutic efficiency of contingency management. It is striking to note that, although formulated using a different theoretical framework, the conclusion drawn here point to cognitive processes similar to those described at the neurobiological level by Baudonnat et al. and Goodman and Packard. Finally, observing the efficiency of eye movement desensitization and reprocessing (EMDR) on posttraumatic stress disorders, an elegant study asked the question of the effects of EMDR on nicotine-related mental imagery and craving (Littel et al.). These intriguing results open an interesting debate about EMDR therapeutic approaches, encouraging future work to determine for how long EMDR-induced improvements may be maintained during protracted abstinence.

In conclusion, the present collection of articles provides original data and new perspectives on a highly promising line of research looking at dynamics of cognitive processes throughout main steps of the addiction cycle, from its initial instatement to treatment.

## Author Contributions

Equal contribution by all the authors.

## Conflict of Interest Statement

The authors declare that the research was conducted in the absence of any commercial or financial relationships that could be construed as a potential conflict of interest.

